# TRPM7‐mediated spontaneous Ca^2+^ entry regulates the proliferation and differentiation of human leukemia cell line K562

**DOI:** 10.14814/phy2.13796

**Published:** 2018-07-23

**Authors:** Kiriko Takahashi, Chisato Umebayashi, Tomohiro Numata, Akira Honda, Jun Ichikawa, Yaopeng Hu, Ken Yamaura, Ryuji Inoue

**Affiliations:** ^1^ Department of Physiology Fukuoka University School of Medicine Fukuoka Japan; ^2^ Department of Anesthesiology Fukuoka University School of Medicine Fukuoka Japan

**Keywords:** ERK‐signaling, erythromyeloid cells, hemoglobin synthesis, spontaneous Ca^2+^ influx

## Abstract

Continuous Ca^2+^ influx is essential to maintain intracellular Ca^2+^ homeostasis and its dysregulation leads to a variety of cellular dysfunctions. In this study, we explored the functional roles of spontaneous Ca^2+^ influx for the proliferation and differentiation of a human erythromyeloid leukemia cell line K562. mRNA/protein expressions were assessed by the real‐time RT‐PCR, western blotting, and immunocytochemical staining. Intracellular Ca^2+^ concentration ([Ca^2+^]_i_) and ionic currents were measured by fluorescent imaging and patch clamping techniques, respectively. Cell counting/viability and colorimetric assays were applied to assess proliferation rate and hemoglobin synthesis, respectively. Elimination of extracellular Ca^2+^ decreased basal [Ca^2+^]_i_ in proliferating K562 cells. Cation channel blockers such as SK&F96365, 2‐APB, Gd^3+^, and FTY720 dose dependently decreased basal [Ca^2+^]_i_. A spontaneously active inward current (*I*_spont_) contributive to basal [Ca^2+^]_i_ was identified by the nystatin‐perforated whole‐cell recording. *I*_spont_ permeated Ca^2+^ comparably to Na^+^, and was greatly eliminated by siRNA targeting TRPM7, a melastatin member of the transient receptor potential (TRP) superfamily. Consistent with these findings, TRPM7 immune reactivity was detected by western blotting, and immunofluorescence representing TRPM7 was found localized to the K562 cell membrane. Strikingly, all these procedures, that is, Ca^2+^ removal, TRPM7 blockers and siRNA‐mediated TRPM7 knockdown significantly retarded the growth and suppressed hemin‐induced *γ*‐globin and hemoglobin syntheses in K562 cells, respectively, both of which appeared associated with the inhibition of ERK activation. These results collectively suggest that spontaneous Ca^2+^ influx through constitutively active TRPM7 channels may critically regulate both proliferative and erythroid differentiation potentials of K562 cells.

## Introduction

Ca^2+^ is a highly versatile intracellular signal that can regulate both acute and long‐term cellular functions ranging from membrane excitation, contraction, neurotransmitter release, secretion, cell growth, differentiation to death (Berridge et al. 2003). In proliferating cells, for instance, a certain level of intracellular Ca^2+^ concentration ([Ca^2+^]_i_) is crucial for the transcriptional/translational processes of cell cycle such as DNA replication and mitosis (Means 1994; Berridge 1995; Whitfield et al. 1995). Thus, disruption of Ca^2+^ homeostasis and dynamics can lead to a variety of cellular pathophysiology in which numerous Ca^2+^‐mobilizing molecules including voltage‐dependent Ca channels, P_2X_, and NMDA inotropic cation channels, Orai and TRP channels play pivotal roles (e.g., Borowiec et al. 2014; Deliot and Constantin 2015).

TRPM7 is a melastatin subfamily member of transient receptor potential (TRP) protein, and has a unique structure dubbed the “chanzyme” which contains both channel and kinase‐like domains (Ryazanova et al. 2004; Visser et al. 2014). TRPM7 channel serves as a constitutively active transmembrane permeant pathway for Ca^2+^ and Mg^2+^, as well as several essential and toxic trace metals (Monteilh‐Zoller et al. 2003). The activity of TRPM7 is effectively regulated by intracellular Mg^2+^ and MgATP levels (Nadler et al. 2001) and modified by phospholipase C‐coupled signaling, endogenous PIP_2_ levels, growth factors, mechanical stress, reactive oxygen species, and extracellular acidity (Aarts et al. 2003; Langeslag et al. 2007; Visser et al. 2014). TRPM7 is expressed in almost all types of cells, and numerous studies have implicated this channel in cell fate‐determining events such as survival, growth, differentiation, and death (Nadler et al. 2001; Abed and Moreau 2007; Visser et al. 2014). Other lines of evidence also suggest close association of TRPM7 with embryonic development, cell morphogenesis/kinesis, inflammatory responses, and tissue remodeling (Clark et al. 2006; Du et al. 2010; Jin et al. 2012; Schappe et al. 2018).

K562 cells were established from a patient with chronic myeloid leukemia and have extensively been used as suitable models to study not only leukaemogenesis but also abnormal haematopoiesis/differentiation into erythrocytic, megakaryocytic and monocytic lineages (Lozzio and Lozzio 1979). Stimulation by hemin (ferriprotoporphyrin IX), sodium butyrate or anthracyclines was shown to differentiate K562 cells to acquire the synthesizing capability of fetal‐form hemoglobin (some part is Ca^2+^ dependent), which thus provides a useful model representing erythropoiesis (Lozzio and Lozzio 1979; Villeval et al. 1983; Tsiftsoglou et al. 2003).

In the present study, we sought the possible role for transmembrane Ca^2+^ influx in regulating proliferation and differentiation of K562 cells, with particular interest in TRPM7. This was prompted because the involvement of Ca^2+^ influx associated with endogenous TRP‐like channels in K562 cells has been suggested for irradiation‐induced cell cycle arrest and consequent survival of K562 cells (Heise et al. 2010). In another study, a Mg^2+^‐dependent cation channel whose properties, however, differ from those of heterologously expressed TRPM7 has been identified in proliferating K562 (Semenova et al. 2005). Moreover, we previously found that the critical contribution of TRPM7‐mediated Ca^2+^ influx to the cell cycle transition at the G1/S boundary in human retinoblastoma cell (Hanano et al. 2004). To facilitate the stable recording of spontaneously active endogenous Ca^2+^ current as well as to better compare with the results obtained by different functional assays, we employed the nystatin‐perforated whole‐cell recording (Horn and Marty 1988) to record TRPM7‐mediated currents by preserving the intracellular milieu as intact as possible. As the result, we have found that continuous Ca^2+^ influx through spontaneously active TRPM7 channel facilitates both proliferation and erythroid differentiation of K562 cells most likely via Ca^2+^‐dependent activation of the ERK signaling.

## Materials and Methods

### Cell culture

A human leukemia cell line K562 was kindly provided by Y. Oyama at Department of Cellular Signaling, Tokushima University. K562 cells were suspended in a 5 mL culture flask containing RPMI1640 (RPMI) medium supplemented with 10% fetal bovine serum at 37°C in an incubator humidified and saturated with 5% CO_2_. Under these conditions, the number of K562 cells doubled per about 2 days, and the medium was changed afresh every 3–4 days.

### Electrophysiology

The equipment and protocols used for patch clamp experiments were essentially the same as those used previously (Hanano et al. 2004). Briefly, a high impedance, low noise patch clamp amplifier (Axopatch 1D, Axon Instruments, Union City, CA; EPC8, HEKA Elektronik, Lambrecht/Pflaz, Germany) in conjunction with an AD,DA converter (TL‐1, Axon Instruments; LIH8+8, InstruTECH, Longmont, CO) was used to apply voltages to and obtain current signals from cells. The obtained signals were low‐pass filtered at 1 kHz and then stored in a computer hard disk after 2 kHz digitization. The computer (Activa, IBM; Dell) was run by the dedicated software (pClamp v.6.03, Axon Instruments; Patchmaster v.2x90.1, HEKA Elektronik). For data analysis and illustration, Clampfit v.9.2 (Molecular Devices, San Jose, CA) and KaleidaGraph v.3.08 (Synergy Software, Reading, PA) were employed depending on the purposes. The input resistance of pipette was 2–4 MΩ when filled with Cs^+^‐based internal solution. About 80–90% of series resistance (5–7 MΩ) was electronically compensated. The liquid junction potential arising at the interface of pipette and external solution (~6 mV) was determined according to the method described by Neher (1992), and corrected when the current–voltage relationship was constructed. For long‐lasting recording (>1 min), MacLab/4 or PowerLab 4/25 (AD Instruments, New South Wales, Australia) was used, with 50 Hz low‐pass filtering and 100 Hz digitization. To minimize measuring errors arising from largely fluctuating currents, they were averaged every 1 sec or longer, and then subjected to analyses.

For nystatin‐perforated recording, nystatin stock solution (Sigma; dissolved in DMSO at 50 mmol/L) was diluted 100–200 times in Cs^+^‐based internal solution, and ultrasonicated for 5–10 min until the aggregates became totally dispersed. In each experiment, a patch electrode was briefly dipped in nystatin‐free internal solution, back‐filled with the one containing nystatin, and tapped with fingers to mix thoroughly. After quickly equipping the pipette to a holder of a patch amplifier, it was pressed onto the cell and the giga‐seal was formed by gentle suction. In a typical time course, the cell membrane was perforated by nystatin within 5 min to lower the access resistance less than 20 MΩ. All experiments were performed at room temperature.

Permeabilities of various external cations relative to Na^+^ through TRPM7‐mediated spontaneously active channels in K562 cells were evaluated under various bi‐ionic conditions by their reversal potentials, the values of which were used to calculate the permeability ratio (*P*_X_/*P*_Na_) according to the modified Goldman–Hodgkin–Katz equation (Lewis 1979):

$$P_{X}/P_{\mathrm{Na}}=\left({\left[{\text{Na}}^{+}\right]}_{o}/{\left[X^{+}\right]}_{o}\right)\exp\left(\Delta E_{\mathrm{rev}}F/RT\right)\;\text{for monovalent cation}\left(*\right)$$

$$\left[\Delta E_{\mathrm{rev}}=E_{\mathrm{rev}(X)}-E_{\mathrm{rev}(\mathrm{Na})}\right]$$

$$\begin{array}{cc} P_{Y}/P_{\mathrm{Na}} & =\left({\left[{\text{Na}}^{+}\right]}_{o}/4{\left[Y^{2+}\right]}_{o}\right)\exp\left(\Delta_{\mathrm{rev}}F/RT\right)\\ & \left[1+\exp\left(E_{\mathrm{rev}(Y)}F/RT\right)\right]\;\text{for divalent cation}\left(*\right) \end{array}$$

$$\left[\Delta E_{\mathrm{rev}}=E_{\mathrm{rev}(Y)}-E_{\mathrm{rev}(\mathrm{Na})}\right]$$

where the activity coefficients of 0.77 and 0.524 were used for monovalent cations and divalent cations (100 mmol/L) respectively. The junction potentials of these solutions to the internal solution were measured separately and corrected.

### Ca^2+^ imaging

After settling on a poly‐l‐lysine‐coated cover slip, K562 cells were treated with fura‐2 am (1* μ*mol/L) for 25–30 min. Global changes in Ca^2+^ concentration ([Ca^2+^]_i_ of these cells were monitored by using a digital fluorescence imaging system (Aquacosmos, Hamamatsu Photonics Co., Shizuoka, Japan). The cell was alternately illuminated by near‐visible ultraviolet excitation lights of 340 and 380 nm and emitted blue lights (filtered at 510 ± 10 nm) were captured through an objective lens of a fluorescence microscope (200× magnification; IX70, Olympus, Tokyo, Japan) by a CCD camera (HISCA, Hamamatsu Photonics) and stored in a computer hard disk. Background fluorescence and autofluorescence from the cell were obtained before fura‐2 loading and subtracted from the fluorescences obtained thereafter. To reduce the influence of quenching, the ratio of fluorescence intensities at 340 and 380 nm excitation (*F*_340_/*F*_380_) was calculated according to the following equation and converted to [Ca^2+^]_i_ values.

$${\left[Ca^{2+}\right]}_{i}+\beta *\left(R-R_{\min}\right)/\left(R_{\max}-R\right)$$

where *R* denotes the ratio (*F*_340_/*F*_380_) of fluorescences at 340 nm and 380 nm (*F*_340_ and *F*_380_, respectively). The values of *β*,* R*_min_, and *R*_max_ were determined in vitro by using a commercial calibration kit (Molecular Probes, Eugene, OR), being 6.29, 0.83, 12.67, respectively.

### Tetracyclin‐inducible siRNA system

In earlier studies, the efficiency of oligonuceotide transfection into nonadherent cells was very low, and in addition, the knockdown of TRPM7 expression was found extremely cytocidal. It was thus practically infeasible to create cell lines stably expressing TRPM7 siRNA. We therefore employed a modified tetracycline‐inducible siRNA expression vector (prototype; pcDNA4/TO; Thermo Fisher Scientific, Waltham, MA) in which RNA‐H1 promoter was inserted to improve the efficiency of siRNA expression (dubbed “pTER^+^”; (van de Wetering et al. 2003)). We then subcloned into this vector the nucleotide sequence corresponding to the 170–188th N‐terminal region of TRPM7 (TM7^170–188^: 5′‐GTCTTGCCATGAAATACTC‐3′) that had previously proved to be an effective target for TRPM7 knockdown (Hanano et al. 2004), which is dubbed as pTER^+^‐TM7‐siRNA. More specifically, hairpin oligonucleotides containing the sense and antisense sequences of TM7^170–188^ respectively (i.e., GATCCC**GTCTTGCCATGAAATACTC**TTCAAGAGA**GAGTATTTCATGGCAAGAC**TTTTTGGAAA and AGCTTTTCCAAAAA**GTCTTGCCATGAAATACTC**TCTCTTGAA**GAGTATTTCATGGCAAGAC**G, respectively) were dissolved at a concentration of 1 *μ*g *μ*L^−1^, 2 *μ*L of which was mixed with 46 *μ*L of annealing buffer (100 mmol/L K‐acetate, 30 mmol/L HEPES‐KOH pH7.4, 2 mmol/L Mg‐acetate), and incubated successively at 90°C for 3 min and at 37°C for 1 h. pTER^+^ was thoroughly digested by the restriction enzymes pair Bgl II/Hind III, electrophoresed on a 1% agarose gel, extracted by using a DNA extraction kit (GE Healthcare Piscataway, NJ), and finally ligated with the hybridized siRNA oligonucleotides by T4 DNA ligase (Thermo Fisher Scientific). The resultant ligation mixture was subjected to transformation, amplification, and ampicillin‐resistant colony selection with DH5*α* competent *Escherichia coli*. Successful incorporation of the siRNA oligonucleotides into the pTER^+^ vector was confirmed by the resistance to EcoR I digestion, the site of which should be deleted after Bgl II/Hind III digestion. The final construct pTER^+^‐TM7‐siRNA was amplified on a large scale with the EndoFree Plasmid Maxi Kit (Qiagen, Hilden, Germany) for later use.

To construct a tetracycline‐inducible expression system in K562 cells, we first introduced the tetracycline repressor (TetR) gene into the K562 genome with an improved electroporation technique (nucleofection; Amaxa BioSystems, Gaithersburg, MD). Briefly, K562 cells were centrifuged, resuspended in a reaction solution containing 2 *μ*g pcDNA6/TR plasmid, and transferred into a cuvette of an electroporator (“Nucleofector”). Both the reaction solution and electroporator were provided by the manufacturer. The optimized protocol for K562 (Nucleofector™ program No.T‐16) was employed to introduce the plasmid into K562 cells. After nucleofection, K562 cells were transferred into a culture flask filled with 10 mL RPMI medium, and incubated for 48 h, and subsequently, 5 *μ*L of 1 mmol/L blasticidin S (Thermo Fisher Scientific) was added to the medium for selection. The amount of blasticidin S was increased in a step‐by‐step manner up to 10 *μ*L per 5 mL. The cells that survived after blasticidin S selection (i.e., TetR^+^) were further transfected by nucleofection with pTER^+^‐TM7‐siRNA plasmid, and 24 h thereafter, selected by adding zeocin (200–400 *μ*g mL^−1^: Thermo Fisher Scientific) in the medium to subclone K562 cells stably expressing both TetR and pTER^+^‐TM7‐siRNA (TetR^+^TM7^+^).

**Figure 1:**
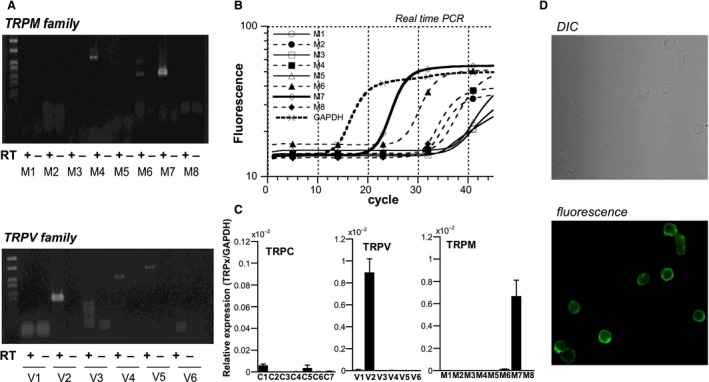
Detection of TRPM7 expression in K562 cells. (A) Conventional RT‐PCR. (B) representative amplification curves of various TRPM isoforms constructed by real‐time PCR. (C) Summary of the results of real‐time PCR (*n* = 5). TRPM7 and TRPV2 are most abundantly expressed among TRP superfamily members. In (B) each amplification curve is plotted according to the calibration calculated for *β*‐actin. The ordinates of histograms in C indicate the relative expression of respective TRP isoforms (normalized to GAPDH expression). (D) Immunocytochemical staining with FITC‐labeled anti‐TRPM7 antibody. DIC image (upper) and immunofluorescence to TRPM7 (lower).

Expression of TRPM7‐siRNA in TetR^+^TM7^+^‐K562 cells was induced by adding doxycycline (0.1, 0.3, 1, 3, 10* μ*mol/L: Sigma) in the culture medium for 96 h or longer. After this treatment, the cells were collected and subjected to RT‐PCR and western blot analyses (Figs. 1 and 5).

### Immunocytochemistry and immnoblotting

#### Immunostaining of TRPM7 protein

Antisera against TRPM7 was raised by immunizing rabbits with a synthesized epitope corresponding to a common amino acid sequence between mouse (1813–1832) and human (1815–1834) TRPM7 C‐termini: CRKLKLPDLKRNDYTPDKII (GenBank accession No. NO_001157797.1 and NP_001288141). K562 cells adherent on cover slips precoated with poly‐L‐lysine were fixed with 4% paraformaldehyde for 15 min and permeablized with 0.2% Triton/PBS for 15 min. After rinsing in PBS several times, the cells were incubated in 10% normal goat serum (Jackson) for 1 h; 1:500 diluted TRPM7 antiserum at 4°C overnight; FITC‐labeled anti‐rabbit goat serum (Jackson Immuno Research Lab. Inc., West Grove, PA) for 1 h. Finally, the cells were embedded in Permafluor aqueous mounting solution (Immunon, Pittsburg, PA). Immunstained K562cells were observed through a laser scanning confocal microscope equipped with an argon/krypton laser source (FV500, Olympus). Emitted light (near 505 nm) by 488 nm excitation was collected as 80 times magnified images and stored in a computer hard disk.

#### Expression analysis by Western Blot

Untreated and treated K562 cells with doxycycline for 96 h were collected. Cell pellets were directly resuspended in sodium dodecyl sulfate (SDS) sample buffer (62.5 mmol/L Tris‐HCl pH 6.8, 2% SDS, 10% glycerol, 50 mmol/L dithiothreitol (DTT), 0.1% bromphenol blue), incubated for 5 min at 95°C, cooled on ice for 5 min and stored at −20°C until further use. The denaturate was then electrophoresed on an acrylamide gel (at 40 mA for 1 h respectively) and blotted on a PVDF membrane. To prevent detecting nonspecific bands, the membrane was shaken in 10%BSA‐containing Tris buffered saline with Tween^®^ 20 (TBS‐T) or phosphate‐buffered saline with Tween^®^ 20 (PBS‐T) for 1 h and rinsed with TBS‐T or PBS‐T; thereafter, allowed to react with either 1:500‐diluted TRPM7 antiserum, 1:2000‐diluted Phospho‐p44/42 MAPK (P‐eErk1/2; 4379, Cell Signaling, Boston, MA), or 1:2000‐diluted p44/42 MAPK (Erk1/2; 4695, Cell Signaling) (in TBS‐T or PBS‐T) at 4°C overnight; subsequently with anti‐rabbit goat IgG (Santa Cruz Biotechnology, Santa Cruz, CA) for 1 h after rinsing with TBS‐T or PBS‐T. After these procedures, the chemiluminescence of the membrane which was treated with the Western Lightning Chemiluminescence Reagent Plus (Perkin Elmer, Boston, MA) was detected by the LAS system (LAS‐3000, Fujifilm, Tokyo, Japan) and analyzed with the Image Gauge software (Fujifilm). *β*‐actin or *α*‐tubulin was taken as a reference, for which mouse monoclonal anti‐*β*‐actin antibody (AC‐15, Abcam, Cambridge, UK), or *α*‐tubulin antibody (T6074, Sigma Aldrich), anti‐mouse IgG, and horseradish peroxidase linked whole antibody (from sheep) (GE Healthcare) were used.

### Reverse transcription polymerase reaction (RT‐PCR) and real‐time PCR

Total RNA (about 1 *μ*g) was extracted from about a million of K562 cells with the total RNA extraction kit (RNeasy Minikit, Qiagen) and reverse transcribed (in a 10 *μ*L scale) by SuperScriptII (Thermo Fisher Scientific) with a randomized or an oligo‐dT primer to obtain the first strand DNA. PCR amplification (in a 20 *μ*L scale) was performed with a heat‐resistant DNA polymerase (PrimeSTAR GXL, Takara) in a thermal cycler (Biometra, Gottingen, Germany) according to the following protocol;denaturation at 94°C for 2 min followed by 17–35 cycles of: denaturation (94°C, 10s), annealing (60°C, 10s), and extension (72°C, 30s). The PCR amplicons were electrophoresed in a 1.2–1.5% agarose gel for 45–60 min, hybridized with ethidium bromide (Nacalai Tesque, Kyoto, Japan) or GelRed™ (Biotium, Fremont, CA), and visualized by ultraviolet or Blue/Green LED light, respectively. The densitometry analysis of electrophoresed PCR amplicons was performed using the ImageJ software (Public domain, NIH, USA).

The sequences of PCR primer pairs are as follows (5′ to 3′, sense/antisense);

TRPM1: GGGGATGCCTTGAAAGACCA/GCCAAGCTCAGCTGATCTGGA

TRPM2: CTTCCGGGAAGGCAAGGATGGT/GAGGCTCACTCCCTGCACGTT

TRPM3: GAGGAGACCATGTCCCCAACTT/GAGTAGCTGTTGGCGCGCT

TRPM4: GTCATCGTGAGCAAGATGATGAA/GTCCACCTTCTGGGACGTGC

TRPM5: CAAGTGTGACATGGTGGCCATCTT/GCTCAGGTGGCTGAGCAGGAT

or GTGACTGTGTTCCTGGGGAA/GACCAGCCAGTTGGCATAGA

TRPM6: GAGGAGATGGATGGGGGCCT/GGTCCAGTGAGAGAAAGCCAACAT

TRPM7: CCATACCATATTCTCCAAGGTTCC/CATTCCTCTTCAGATCTGGAAGTT

TRPM8: GAAGGCACCCAGATCAACCAAA/GAGCCTTCCACCACCACACA

or CTTCGTGGTCTTCTCCTGGAA/CATGGCCAGGTAGGGCTC

GAPDH: ATCACCATCTTCCAGGAGCGAG/TGGCATGGACTGTGGTCATG

or GGTGAAGGTCGGAGTCAACG/CAAAGTTGTCATGGATGACC

*γ*‐globin: GGCAACCTGTCCTCTGCCTC/GAAATGGATTGCCAAAACGG

In separate experiments, quantitative real‐time PCR was performed for all other TRP superfamily members in addition to those of TRPM subfamily with primer pairs; (5′ to 3′; sense/antisense).

TRPC1: GCGTAGATGTGCTTGGGAGAAA/GCTCTCAGAATTGGATCCTCCTCT

TRPC2: GCTGGCCAAGCTGGCCAA/CATCCTCACTGGCCAGCGAGA

TRPC3: CCTCTCAGCACATCGACAGGT/GAACACAAGCAGACCCAGGAAGA

TRPC4: CAAGCTTCTAACCTGCATGACCA/CCAAATATTGACCAAAACAGGGA

TRPC5: CATCCCAGTGGTGCGGAAGA/CCTAAGTGGGAGTTGGCTGTGAA

TRPC6: GAGGAGGAGCGCTTCCTGGACT/GCCTTCAAATCTGTCAGCTGCA

TRPC7: CCAGGTGGTCCTCTGCGGAA/GGCTCAGACTTGGACGGTGGT

TRPV1: GAAGATCGGGGTCTTGGCCTA/CTCACTGTAGCTGTCCACAAACAAA

TRPV2: GACGTGCCTGATGAAGGCTGT/CTGGTGTGGGTTCTCCAGGA

TRPV3; AGTGGCAACTGGGAGCTGG/GGGTCAGGGTGATGTTGTAGAAGA

TRPV4: GTGCCTGGGCCCAAGAAA/GGGCAGCTCCCCAAAGTAGAA

TRPV5: CTCACCCCCTTCAAGCTGGCT/CCCAGCATCTGGAATCCTCG

TRPV6: GCCGAGATGAGCAGAACCTGCT/GTCTGGTCCAGGATCTGGCGA

Real‐time PCR was implemented by using the “Line‐Gene” Fluorescent Quantitative Detection System (BioFlux, Tokyo, Japan)or Smart Cycler System II (Cepheid, Sunnyvale, CA)with the first strand DNA and PCR reagent (TakaraBio Inc., Shiga, Japan or TOYOBO, Osaka, Japan) according to the manufacturer's instructions. Data analysis was made with the software dedicated to the respective systems.

### Cell counting assay

K562 cells (5x10^4^) were replated in a 6‐cm dish and incubated in Ca^2+^‐free, 10% serum‐added RPMI medium for 3 days with or without 10 *μ*g mL^−1^ doxycycline. Thereafter, an aliquot (1 *μ*L) of the Acridine Orange/Propidium Iodide (AO/PI) Cell Viability Kit (Logos Biosystems, Republic of Korea) was added to each 100 *μ*L of samples. After the incubation at room temperature for 10 min, the cell staining solution was loaded onto the counting slide of Countess and the loaded cell sample images were acquired from CountessII‐FL (Thermo Fisher Scientific). The cells positive for AO were taken viable and counted to calculate the proliferation rate.

### Quantitation of hemoglobin synthesis

To assess hemoglobin synthesis, K562 cells were stimulated with hemin (40* μ*mol/L) for 3 days, and then centrifuged and washed with PBS. The resultant cell pellet was resuspended in lysis buffer (100 mmol/L potassium phosphate pH 7.8, 0.2% Triton X‐100) and incubated 10 min at room temperature. After precipitating cellular debris by centrifugation, the supernatant was collected and the quantity of hemoglobin contained in it was determined using the plasma hemoglobin colorimetric assay kit, according to the manufacturer's instructions (Cayman Chemical, Ann. Arbor, MI). The hemoglobin concentration was calculated as g dL^−1^ by dividing the Hb quantity by the number of cells measured by the Countess II‐FL (Thermo Fisher Scientific).

### Solutions

The composition of solutions used were as follows.

**Figure 2:**
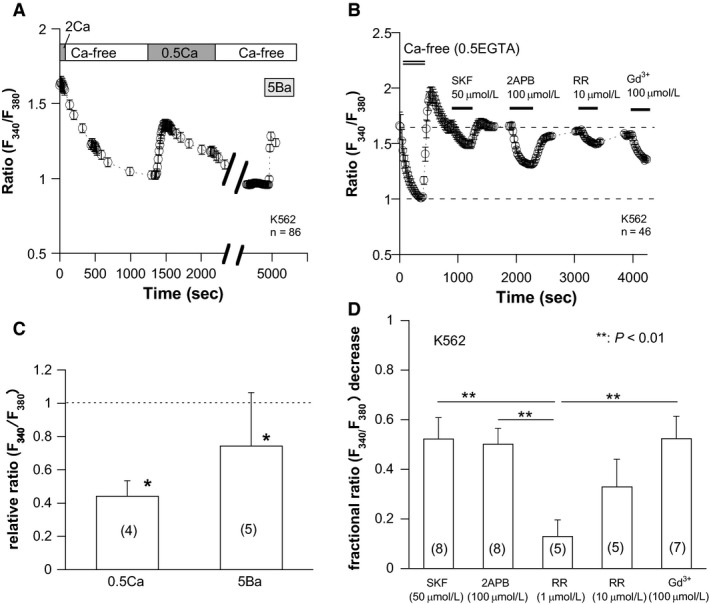
Ionic and pharmacological properties of basal Ca^2+^ influx in K562 cells. Fura‐2 fluorescence imaging. (A) switching from PSS (2 mmol/L Ca^2+^ present: 2Ca) to Ca^2+^‐free PSS lowered basal Ca^2+^ level, and subsequent application of 0.5 mmol/L Ca^2+^ (0.5Ca) or 5 mmol/L Ba^2+^ (5Ba) partially recovered it. (B) SK&F96365 (SKF), 2‐aminoethoxydiphenyl borate (2‐APB), ruthenium red (RR), and Gd^3+^ effectively inhibited basal Ca^2+^ influx at the concentrations indicated. Note: in A, “Ca‐free” denotes nominally Ca^2+^‐free external solution, while in B, “Ca‐free (0.5EGTA)” denotes that 0.5 mmol/L EGTA is added in Ca‐free external solution. The numbers of cells simultaneously tested (*n*) are shown in panel A and B. (C) Changes in the fluorescence ratio (*F*_340_/*F*_380_) by switching from Ca‐free to 0.5 mmol/L Ca^2+^ (0.5Ca) or 5 mmol/L Ba^2+^ (5Ba)‐containing bath solutions are normalized to the ratio difference between Ca‐free and 2 mmol/L Ca‐containing bath solutions as shown in A, and averaged. Fluorescence changes between Ca‐free and 0.5 mmol/L Ca^2+^‐ or 5 mmol/L Ba^2+^‐added conditions are statistically significant (*) by paired t‐test (*P *<* *0.05). (D) fractional reductions of *F*_340_/*F*_380_ ratio (normalized to the ratio difference between Ca‐free and 2 mmol/L Ca‐containing bath solutions) after application of drugs (for abbreviations, see above). Data obtained from experiments such as shown in B are averaged. The numbers in parentheses in C and D denote those of independent imaging experiments, each being derived from the same batches of 17–86 K562 cells. ** indicates *P *<* *0.01 with Tukey's multiple comparison test. Only statistically significant results are shown.

Extracellular (bath) solution (physiological saline solution: PSS) (in mmol/L): 140 Na^+^, 5 K^+^, 1.2 Mg^2+^, 2 Ca^2+^, 151.4 Cl^‐^, 5 glucose,10 HEPES (adjusted at pH7.4 with Tris^+^). Drugs were dissolved in PSS and topically applied through a home‐made, fast solution change device (solenoid‐valve driven “Y‐tube”). To prepare Ca^2+^‐deficient external solution, 0.5 mmol/L EGTA was added to thoroughly chelate residual Ca^2+^. In some experiments measuring Ba^2+^ fluorescence (Fig. 2A and C), nominally Ca^2+^‐free rather than 0.5 mmol/L EGTA‐containing external solution was used.

Ca^2+^, Mg^2+^‐free Na^+^ external solution consisted of (mmol/L): 150 Na^+^, 150 Cl^‐^, 1 EDTA, 5 glucose, 10HEPES; Ca^2+^, Ba^2+^, or Mg^2+^ external solutions (mmol/L);100 Ca^2+^, Ba^2+^, or Mg^2+^, 200 Cl^‐^, 5 glucose, 10 HEPES (adjusted at pH7.4 with Tris^+^).

Cs‐based internal solution for conventional and nystatin‐perforated whole‐cell recordings contained (mmol/L):140 Cs^+^, 20 Cl^‐^, 120 aspartate^‐^, 5BAPTA, 1.5Ca^2+^, 10 glucose, 10HEPES (adjusted at pH7.4 with Tris^+^).

### Material and drugs

N‐methyl, D‐glucamine (NMDG), adenosine 1,4,5‐trisphosphate (ATP), 1,2‐bis(o‐aminophenoxy)etane‐N,N,N′,N′‐tetraacetic acid (BAPTA), 1‐(4‐aminobenzyl) ethylenediamine‐N,N,N′,N′‐tetra acetic acid (AM‐EDTA), ethyleneglycol‐bis(*β*‐aminoethyl)‐ N,N,N′,N′‐ tetra acetic acid (EGTA), ethylenediamine‐N,N,N′,N′‐tetra acetic acid (EDTA), hemin, HEPES, and GdCl_3_ were purchased from Sigma and SK&F96365, 2‐aminoethoxydiphenyl borate (2‐APB) and ruthenium red (RR) from Calbiochem, and FTY720 from Cayman Chemical, respectively.

pTER^+^ was kindly provided by Dr. M. van de Wetering (Hubrecht Laboratory, Center for Biomedical Genetics, Netherlands).

### Statistical evaluation

All data shown in figures are expressed as mean ± SEM. Statistical significance between different groups was assessed by Student's paired or unpaired *t*‐test for single comparison and two‐way ANOVA followed by Tukey's test for multiple comparison with the aid of a commercial software JMP v.12.2 (SAS Institute Inc., Cary, NC).

## Results

### TRPM7 is a predominant TRP isoform expressed in K562

Both conventional and quantitative real‐time PCR experiments in K562 cells indicated that TRPM7 and TRPV2 are most predominantly expressed isoforms among all TRP family members (Fig. 1A–C). Immunostaining of K562 cells with anti‐TRPM7 antibody confirmed robust expression of TRPM7 protein, which is largely confined to the cell membrane (Fig. 1D; see also Fig. 5C).

Widespread expression of TRPV2 has been recognized in both myeloid and lymphoid leukemia cells (Morelli et al. 2013). However, no such evidence has been obtained for TRPM7 despite its postulated ubiquity. Thus, TRPM7 may be an abundant TRP isoform uniquely expressed in K562 cell.

### K562 possesses robust basal Ca^2+^ influx

TRPM7 is known as a spontaneously active channel permeant to Ca^2+^ and Mg^2+^ near the resting membrane potential (Nadler et al. 2001). Thus, it may act as a constitutive entry pathway for Ca^2+^ and other divalent cations in K562 cells. Consistent with this expectation, removal of external Ca^2+^ remarkably lowered the intracellular Ca^2+^ concentration ([Ca^2+^]_i_) of K562, when monitored by fura‐2 fluorescent imaging technique (Fig. 2A). Replacement of Ca^2+^ with Ba^2+^ partially restored fura‐2 fluorescence (Fig. 2A and C). In addition, the basal [Ca^2+^]_i_ level of K562 cells was reversibly reduced by nonspecific cation channel blockers including SK&F96365 (SKF), 2‐APB, ruthenium red (RR), and Gd^3+^ at 10–100 *μ*mol/L concentrations (Fig. 2B and D). Notably, ruthenium red only slightly (by ~10%) reduced the basal [Ca^2+^]_i_ level at the concentration (1 *μ*mol/L) reported to almost completely inhibit endogenous TRPV2 channels (Fig. 2D; (Pottosin et al. 2015)). This suggests that these channels may contribute only marginally to basal Ca^2+^ influx in K562 cells.

### Spontaneously active inward current in K562

**Figure 3:**
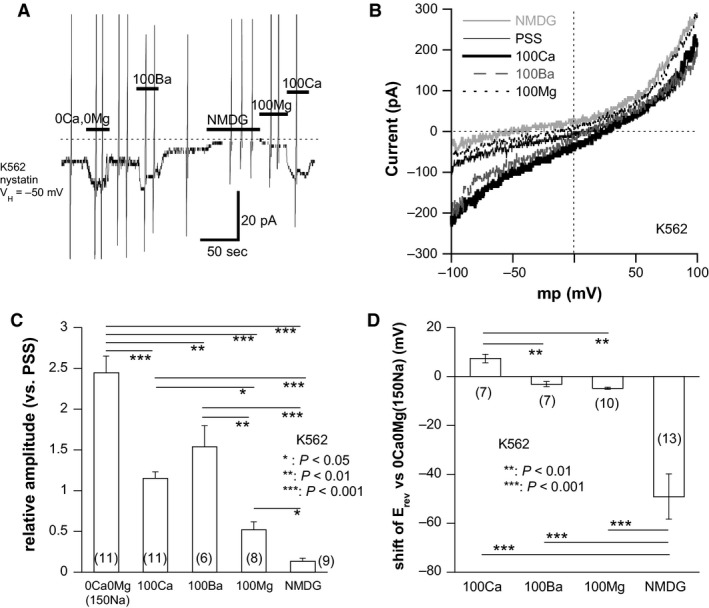
Spontaneous inward current (*I*_spont_) and its divalent cation permeability in K562 cells. (A) An actual trace of *I*_spont_ recorded from a K562 cell by the nystatin‐perforated whole‐cell patch clamp (holding: −50 mV). Bath solution: PSS (2 mmol/L Ca^2+^ present). Pipette solution: Cs^+^‐based internal solution. At respective bars, the following solutions were rapidly applied through the “Y‐tube” device; NMDG (150 mmol/L NMDG‐Cl); 100Mg (100 mmol/L MgCl_2_); 100Ca (100 mmol/L CaCl_2_); 100Ba (100 mmol/L BaCl_2_); 0Ca0Mg (150 mmol/L Na without divalent cations). Vertical deflections indicate the currents induced by ascending ramp voltages (−120 to +100 mV, 1 sec). (B) representative current–voltage (mp) relationships of *I*_spont_ evaluated by ramp voltages under various ionic conditions as shown in A. (C) fold changes of *I*_spont_ amplitude under various ionic conditions as shown in A. The amplitudes of *I*_spont_ under various ionic conditions (0Ca0Mg, 100Ca, 100Ba or NMDG) are normalized to that in PSS, and averaged. (D) averaged shifts of the reversal potential (*E*_rev_) of *I*_spont_ under various ionic conditions relative to the *E*_rev_ in 0Ca0Mg (i.e., 150 mmol/L NaCl) are shown. The numbers in parentheses in C and D denote those of independent K562 cells tested. *, **, and *** respectively indicate *P* values of <0.05, <0.01 and <0.001 with Tukey's multiple comparison test. Only statistically significant results are shown.

**Figure 4:**
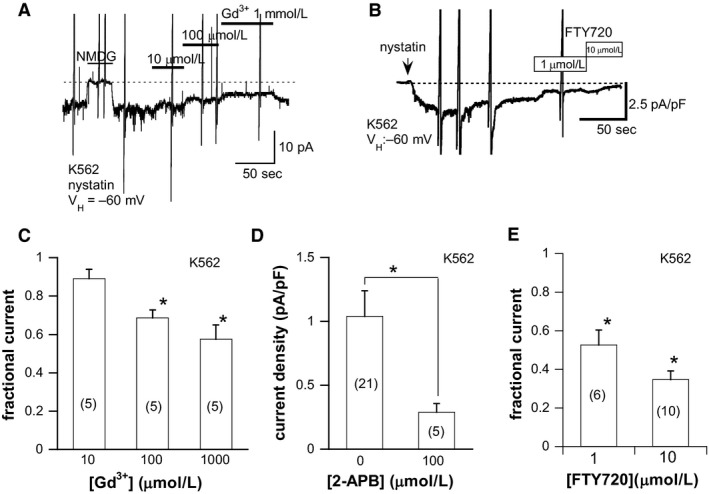
Pharmacological properties of *I*_spont_ in K562 cells. (A, B) Actual traces showing the inhibitions of *I*_spont_ by TRPM7 channel blockers, Gd^3+^, and FTY720, respectively. The recording conditions were the same as in Figure 3. (C) concentration‐dependent inhibition of *I*_spont_ by 10, 100, and 1000* μ*mol/L Gd^3+^. The fraction of *I*_spont_ remaining after the inhibition (fractional current) is averaged and illustrated. (D) inhibitory effects of 2‐APB on the current density of *I*_spont_. (E) relative inhibitions of *I*_spont_ by FTY720 at 1 and 10* μ*mol/L. The fraction of *I*_spont_ remaining after the inhibition (fractional current) is averaged and illustrated. The numbers in parentheses indicate the numbers of K562 cells tested. *P <* *0.05 with Dunnett’ test versus control (i.e., before application of drugs) (C and E) or unpaired Student *t*‐test (D).

To confirm that external Ca^2+^‐dependence of basal [Ca^2+^]_i_ observed above reflects the Ca^2+^ influx through spontaneously active TRPM7‐channels, we next conducted a nystatin‐perforated whole‐cell recording in K562 cells under the conditions that would minimally disturb the intracellular homeostasis and metabolism. As demonstrated in Figure 3A, a small but robust inward‐going current (*I*_spont_) was recorded at −50 or −60 mV without stimulation. The magnitude of *I*_spont_ was doubled in the absence of Ca^2+^ and Mg^2+^ (i.e., Na^+^ is the sole charge carrier) and totally abrogated when all cations were substituted by a large membrane‐impermeant cation N‐methyl, d‐glucamine (NMDG). Furthermore, isotonic substitution of external cations with Ca^2+^, Mg^2+^, or Ba^2+^ also allowed measurable inward currents (Fig. 3C). The current–voltage (*I*–*V*) relationships of *I*_spont_ under various ionic conditions evaluated by a slow rising ramp voltage showed prominent outward rectification with distinguishable shifts of the reversal potentials for Ca^2+^, Mg^2+^, and Ba^2+^ (Fig. 3B and D). The relative permeability ratios of Ca^2+^, Ba^2+^, and Mg^2+^ to Na^+^ (calculated from these shifts; Fig. 3D) are 1.21 (±0.15, *n* = 7), 0.77 (±0.08, *n* = 7), and 0.63 (±0.01, *n* = 10), respectively. Although estimation of the cation permeability based on this method could be somewhat inaccurate with discrepancies to previously reported values, the high Ca^2+^ permeability is a commonly observed feature of TRPM7 channel (Monteilh‐Zoller et al. 2003; Hanano et al. 2004; Li et al. 2006). In support of this view, known blockers of TRPM7 such as Gd^3+^, 2‐APB, and FTY720 (which inhibits TRPM7 relatively selectively) (Li et al. 2006; Qin et al. 2013; Chubanov et al. 2014), dose‐dependently inhibited *I*_spont_ (Fig. 4).

**Figure 5:**
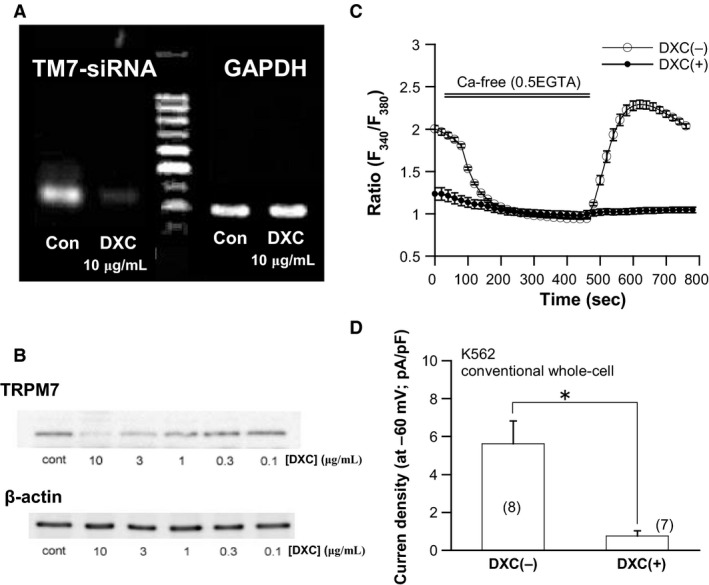
siRNA knockdown of TRPM7 channel abrogates Ca^2+^ influx and *I*_spont_ in K562 cells. (A) detection of TRPM7 transcripts (398 bp) by conventional RT‐PCR. Ten *μ*mol/L doxycycline (DXC) almost completely eliminated TRPM7 mRNA expression. (B) immunoblots of K562 protein extracts by TRPM7 antibody. Doxycycline concentration dependently (0.1–10* μ*mol/L) reduced TRPM7 protein expression. The results shown in A and B are representative of three independent experiments. (C) fura‐2 Ca^2+^ fluorescence imaging from untreated ([DXC(−)] and 10* μ*mol/L doxycycline‐treated [DXC(+)] K562 cells stably expressing *tet*‐inducible TRPM7‐specific siRNA (siTRPM7). *n* = 36 and 26, respectively. (D) density of *I*_spont_ recorded by conventional whole‐cell recording from untreated ([DXC(−)] and 10* μ*mol/L doxycycline‐treated [DXC(+)] K562 cells stably expressing siTRPM7. To facilitate the induction of TRPM7 currents, ATP and Mg^2+^ were absent in the patch pipette. The numbers of cells tested are shown in parentheses.

To more rigorously verify that the above observed *I*_spont_ reflects endogenous TRPM7 channel activities, we next adopted the siRNA strategy specifically targeting TRPM7 (see the Methods). After substantial knockdown of TRPM7 expression by its specific siRNA induced by doxycycline [DXC(+); Fig. 5A and B], basal [Ca^2+^]_i_ was much lower than control [i.e., without doxycycline; DXC(−)] and removal of Ca^2+^ from the bath produced only a marginal decrease in [Ca^2+^]_i_ (filled circles in Fig. 5C). Whole‐cell recording also confirmed this observation with significantly reduced inward current density after TRPM7 knockdown (Fig. 5D).

These results strongly suggest that both basal [Ca^2+^]_i_ and *I*_spont_ reflect endogenous TRPM7 channel activities.

### TRPM7 activity is functionally linked to K562 proliferation and erythroid differentiation

**Figure 6:**
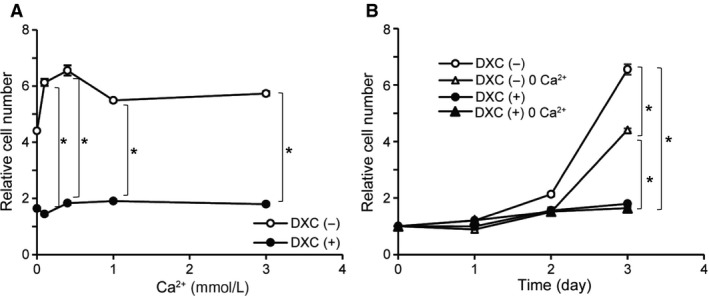
K562 cell growth is dependent on Ca^2+^ influx mediated by TRPM7 channel. (A) Dependency of K562 cell growth on extracellular Ca^2+^ concentration evaluated at 72 h after culture. Open and filled circles indicate K562 cells untreated [DXC(‐)] or treated with 10* μ*mol/L doxycycline [DXC(+)], respectively. (B) time courses of K562 cell growth under various conditions, i.e. DXC(−) (no doxycycline and normal Ca^2+^ in RPMI medium), DXC(−)0Ca^2+^ (no doxycycline and Ca^2+^ free in the medium), *DXC(+)* (10* μ*mol/L doxycycline and normal Ca^2+^ in the medium) and DXC(+)0Ca^2+^ (10* μ*mol/L doxycycline and Ca^2+^ free in the medium). In both types of experiments (A and B), K562 cells stably expressing *tet*‐inducible TRPM7‐siRNA (siTRPM7) were used. *n* = 5 for each condition. *P <* *0.05 with two‐way ANOVA with repeated measures and Tukey's multiple comparison test. To avoid promiscuity, the results of statistical evaluation are shown only for selected pairs of groups.

In the final step, we explored the functional linkage of TRPM7 activity to the proliferation and erythroid differentiation of K562 cells. As shown in Figure 6A and B, the growth rate of K562 was dependent on external Ca^2+^ concentration and significantly slowed down in the absence of Ca^2+^ in the culture medium [open circles in Fig. 6A; DXC(−)0Ca in Fig. 6B]. Strikingly, the proliferation of K562 almost completely ceased after siRNA knockdown of TRPM7 expression, which was not rescued by increasing the extracellular Ca^2+^ concentration [filled circles in Fig. 6A; DXC(+) in Fig. 6B]. These results strongly suggest that basal Ca^2+^ influx is essential for K562 cell growth, the major part of which likely occurs through spontaneously active TRPM7 channels.

**Figure 7:**
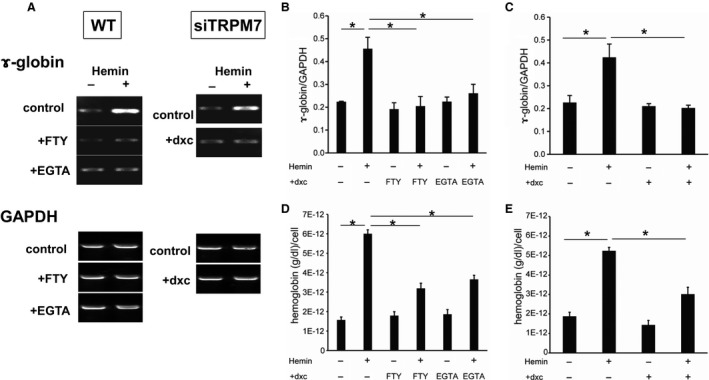
Erythroid differentiation of K562 cell by hemin is regulated by Ca^2+^ influx mediated by TRPM7. (A) representative RT‐PCR experiments showing hemin‐induced *γ*‐globin synthesis in K562 cells untreated (control) and treated with 10* μ*mol/L FTY720 (+FTY), 1 mmol/L EGTA (+EGTA) or 10* μ*mol/L doxycycline (+dxc). In all conditions, K562 cells stably expressing *tet*‐inducible TRPM7‐siRNA (siTRPM7) were used. The expression of *γ*‐globin mRNA was greatly decreased by these procedues, while that of GAPDH stayed almost constant. (B and C) statistical evaluation of pooled data from experiments as shown in A: 10* μ*mol/L FTY720 and 1 mmol/L EGTA (B): siTRPM7 (C). (D and E) effects of 10* μ*mol/L FTY720 (FTY), 1 mmol/L EGTA (EGTA) or 10* μ*mol/L doxycycline (+dxc) on hemoglobin synthesis at rest or 3 days after hemin treatment in K562‐cells stably expressing siTRPM7. *n* = 5 for each condition. *P <* *0.05 with two‐way ANOVA and Tukey's multiple comparison test. To avoid promiscuity, the results of statistical evaluation are shown only for selected pairs of groups.

In a different series of experiments, we also investigated the possible contribution of TRPM7 activity to the differentiation of K562 cells into an erythroid lineage. As well known, many reagents triggering erythropoiesis can induce the synthesis of a fetal form of hemoglobin in K562 cells (see the Introduction). We used hemin as a potent erythropoietic reagent and measured *γ*‐globin as the differentiation marker, the essential subunit of fetal hemoglobin (Dean et al. 1983). As shown in Figures 7A–C, 24‐h treatment of K562 cells with hemin remarkably enhanced the expression of *γ*‐globin without affecting TRPM7 activities (Fig. S1). Importantly, siRNA knockdown of TRPM7 expression, and chelation of Ca^2+^ by EGTA or addition of the TRPM7 blocker FTY720 in the culture medium all abolished hemin‐induced *γ*‐globin synthesis in K562 cells. Impairment of erythroid differentiation by these procedures was also confirmed by direct determination of hemoglobin concentration (Fig. 7D and E).

**Figure 8:**
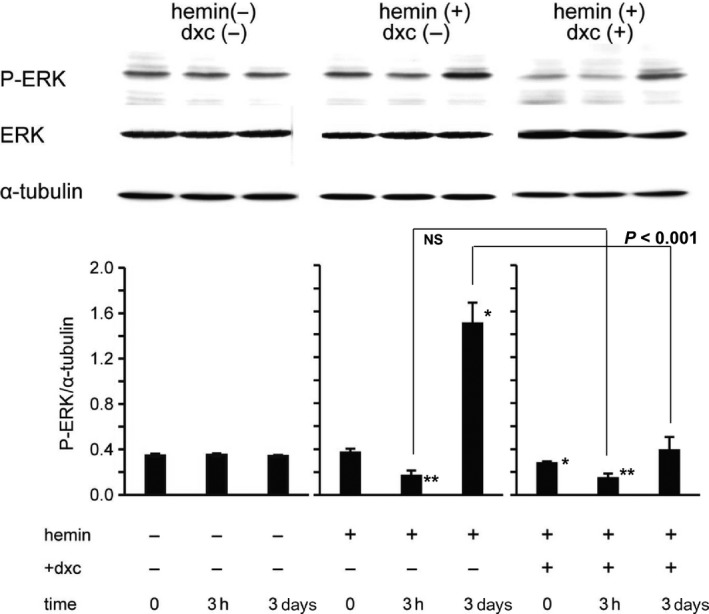
Time‐dependent ERK phosphorylation under hemin stimulation without or with siTRPM7 treatment. The respective time courses of phosphorylated ERK, ERK and *α*‐tubulin protein expressions. Assessed by the immunoblotting technique with siTRPM7‐expressing K562 cells untreated [hemin(−), dxc(−)] or treated with hemin [(hemin(+)], and/or doxycycline (+dxc) before (0 h) and 3 h or 3 days after the start of each experimental condition. Upper and lower panels indicate actual results and their relative expression levels (normalized to *α*‐tubulin protein expression which did not change during the course of experiments) averaged from five independent experiments, respectively. *, and ** respectively indicate *P* values of <0.05 and <0.01 versus the corresponding time controls (three leftmost columns) with Tukey's multiple comparison test. The results of Tukey's test for the columns paired by *U*‐shaped lines are also shown.

Previous studies reported that the ERK signaling is crucial for both K562 proliferation and differentiation (Whalen et al. 1997; Kang et al. 1999; Witt et al. 2000; Sawafuji et al. 2003; Woessmann et al. 2004; Kucukkaya et al. 2006). We therefore tested whether this signaling is involved in the observed differentiation of K562. As demonstrated in Figure 8, in response to hemin, the extent of ERK phosphorylation declined in hours but increased subsequently (in days). This observation is consistent with the finding of a previous study (Woessmann et al. 2004). Notably, the knockdown of TRPM7 attenuated the extent ERK phosphorylation not only in prior to hemin treatment, but also at the late phase (3 days) after hemin treatment [DXC(+) in Fig. 8], suggesting that ERK activation may be involved in both proliferation and differentiation of K562 cells.

## Discussion

The present study has clearly shown that K562 cells constitutively express TRPM7 channels which likely serve as a spontaneously active Ca^2+^ entry pathway essential for maintaining basal [Ca^2+^]_i_. This is evidenced by a few key observations, that is, robust expressions of TRPM7 mRNA and protein and identification of associated Ca^2+^‐transporting activities in K562 cells, and their effective elimination by treating with TRPM7‐targeting siRNA or applying TRPM7 blockers such as FTY720. Importantly, the procedures compromising the Ca^2+^ influx through this channel, that is, its blockers, siRNA knockdown, or removal of extracellular Ca^2+^, all resulted in pronounced inhibition of both proliferation and erythroid differentiation by hemin. These results strongly suggest that a certain basal [Ca^2+^]_i_ level is a prerequisite to the proliferation and differentiation of K562 cells, to which TRPM7‐mediated Ca^2+^ influx indispensably contributes. Indeed, many other studies have reported that Ca^2+^ homeostasis maintained by basal Ca^2+^ influxes through various cell‐specific Ca^2+^‐permeable channels are crucial for cellular functions and dysfunctions (Berridge et al. 2003; Bagur and Hajnoczky 2017).

The differentiation of erythromyeloid cells is regarded as a highly coordinated set of numerous signaling events which should occur in the right order at the right timing. A plethora of transcriptional regulators such as transcription factors, kinases, noncoding RNAs, and DNA‐binding proteins are involved therein (Tsiftsoglou et al. 2003; Hattangadi et al. 2011). There is good evidence that some steps of it depend critically on preceding elevations in [Ca^2+^]_i_ or the presence of Ca^2+^ in the external milieu (Levenson et al. 1980; Tsiftsoglou et al. 1981). In apparent agreement, it is known that K562 cells express Ca^2+^‐mobilizing G protein‐coupled receptors whose stimulation lead to [Ca^2+^]_i_ elevation (Thomas et al. 1995). However, other studies provided seemingly contradictory observations. Albeit indirect evidence, it was shown that downregulation of a voltage‐dependent Ca^2+^ channel gene *Cacnad1* by mircoRNA‐107 rather promotes K562 erythroid differentiation (Ruan et al. 2015). A hERG channel toxin BmKKx2, which is expected to depolarize the cell membrane, thereby reducing the driving force for transmembrane Ca^2+^ influx, was shown to retard the proliferation and facilitate the AraC‐induced erythroid differentiation of K562 cells, respectively (Zhang et al. 2007; Ma et al. 2013). Furthermore, stimulation of Ca^2+^‐mobilizing glutamate receptor was found to enhance the proliferation of erythromyeloid stem cells including Meg‐01, Set‐2, and K562 cells, while its antagonist facilitated Meg‐01 differentiation to megakaryocytes (Kamal et al. 2015). These results are consistent with the view that reduction of basal [Ca^2+^]_i_ is a crucial event to cease proliferation and then initiate differentiating processes. In fact, our present study has also confirmed that the growth of K562 cells depended on the presence of extracellular Ca^2+^, and interventions to decrease [Ca^2+^]_i_ significantly decelerated it (Fig. 6A and B). Nevertheless, the reduction of [Ca^2+^]_i_ also impaired the hemoglobin synthesis induced by hemin (Fig. 7). These confounding results may imply the involvement of inexplicably intricate Ca^2+^‐dependent mechanisms in the control of proliferation and differentiation. In this regard, one reconciling explanation could be that ERK activation follows variable, multi‐phasic time courses in response to differentiating agents ((Woessmann et al. 2004); see below). It is well recognized that the Ras‐ERK signaling occupies a central part in regulating cell proliferation and differentiation, where Ca^2+^‐dependent activation of Ras (e.g., through Ca^2+^/calmodulin‐mediated regulation of Ras‐guanine nucleotide‐releasing factor or via complex Pyk2‐mediated signaling) may play a pivotal role (Cullen and Lockyer 2002).

In erythrogenesis, there are disparate findings that both ERK activation and inhibition can promote differentiation of stem/progenitor cells into matured erythroid cells capable of producing hemoglobin. For example, while pharmacological inhibition of the Ras–Raf–Mek1–ERK signaling inhibits K562 proliferation leading to its erythroid differentiation, enhanced ERK activity by Ras overexpression is also shown to promote the differentiation (Woessmann et al. 2004). Woessmann et al. (2004) investigated in detail this paradoxical commitment of ERK to erythroid differentiation in K562 cells by careful chasing the time courses of ERK phosphorylation and concomitant hemoglobin synthesis. Their key findings are recapitulated as follows; in response to erythroid‐differentiating agents, ERK phosphorylation declines in hours but thereafter follows differential time courses dependent on the differentiating agents used. While the phosphorylation remained decreased with butyrate or Ara‐C, it increased again more than 24 h later with cisplatin or hemin as the differentiating agent, and the late reactivation of ERK by cisplatin or hemin occurred at the same timing as the induction of hemoglobin synthesis. Moreover, MEK‐1 inhibitors which decrease ERK phosphorylation induced the erythroid differentiation of K562 cells as well as inhibited hemin‐ or cisplatin‐induced hemoglobin synthesis. The most plausible interpretation given to these findings is that inhibition of ERK activity is commonly involved in the initiation of erythroid differentiation, in other words, cessation of proliferation of K562 cells. In contrast, reactivation of ERK may be crucial for further forwarding the erythrogenesis by hemin or cisplatin. Our immunoblotting results on ERK phosphorylation exactly match up with this view (Fig. 8). In addition, it should be emphasized that TRPM7 channel activities (i.e., Ca^2+^ influx thereby) are essential to maintain the ERK activity both before and after hemin stimulation, respectively (Fig. 8), so as to drive the proliferation and erythroid differentiation of K562 cells (Figs. 6 and 7). This is the most important conclusion of the present study. At present, how such Ca^2+^ influx via TRPM7 channel would precisely divert the ERK‐mediated signaling to either proliferation or differentiation remains unclear. Thus, it will need to be elucidated, in particular regarding the possible involvement of the Ras‐GRP or Pyk2‐mediated signaling (Cullen and Lockyer 2002).

In summary, the present study has unveiled previously unrecognized Ca^2+^‐dependent mechanisms via spontaneously active TRPM7 channels that likely regulate not only the proliferative potential but hemin‐induced erythroid differentiation of a human erythromyeloid leukemia cell line K562, in which ERK activation with different time courses may be involved. These results will further point to the possibility that any defective gating of TRPM7 channel due to hereditary and/or acquired causes might lead to human hematopoietic disorders such as anaemia and thrombocytopenia.

## Conflict of Interest

None.

## Data Accessibility

## Supporting information




**Figure S1.** Hemin treatment does not affect TRPM7 activity.
