# Supplementary figures and images for: TRPM7‐mediated spontaneous Ca2+ entry regulates the proliferation and differentiation of human leukemia cell line K562
Source: Physiol Rep. 2018 Jul 23;6(14):e13796. doi: 10.14814/phy2.13796 (PMC6055029; doi:10.14814/phy2.13796)

## Supplementary Figure 1

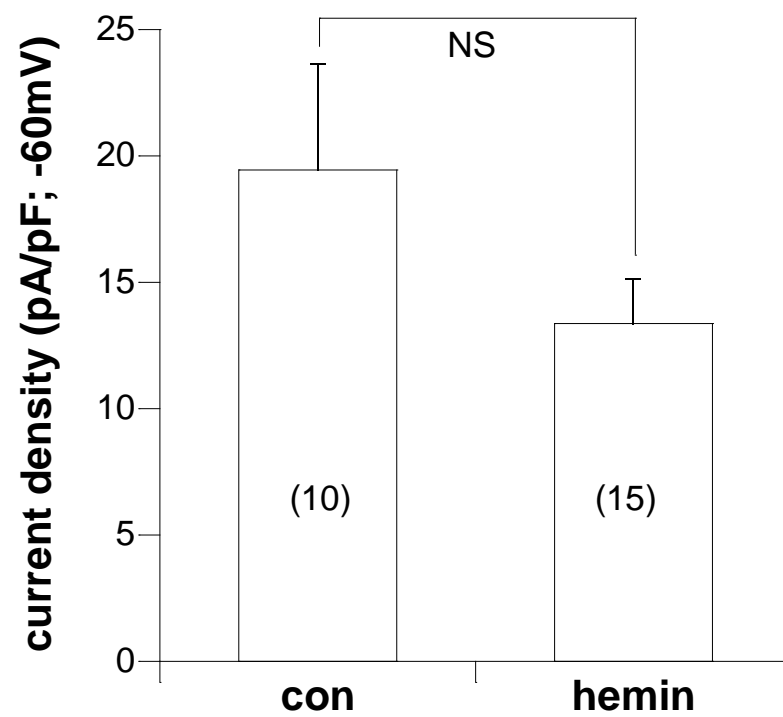

Supplement: Supplementary file 1 — Figure S1. Hemin treatment does not affect TRPM7 activity. [file PHY2-6-e13796-s001.pdf]
