# Supplementary material for: TRPM7‐mediated spontaneous Ca2+ entry regulates the proliferation and differentiation of human leukemia cell line K562
Source: Physiol Rep. 2018 Jul 23;6(14):e13796. doi: 10.14814/phy2.13796 (PMC6055029; doi:10.14814/phy2.13796)
Supplement: Supplementary file 2 [file PHY2-6-e13796-s002.docx]

Supplementary Fig. 1

Hemin treatment does not affect TRPM7 activity.

TRPM7-mediated currents were measured by introducing Cs-based, ATP-free, Mg^2+^-free internal solution (see the Methods) into non-treated or hemin (40μM, 3days)-treated K562 cells at a holding potential of -60mV. Data are expressed as the averaged current density (pA/pF) at -60mV after normalizing to the cell capacitance. The numbers in parentheses indicate those of independent measurements. There is no statistically significant difference between non-treated and hemin-treated K562 cells with unpaired student t-test.
